# Pneumococcal vaccination in older persons: where are we today?

**DOI:** 10.1186/s41479-017-0045-y

**Published:** 2018-01-05

**Authors:** Paul Van Buynder, Robert Booy

**Affiliations:** 1 Griffith University, Southport, QLD Australia; 2 Immunisation Coalition, Melbourne, VIC Australia; 3 Sydney University, Sydney, Australia

**Keywords:** Pneumococcus, Vaccination, Effectiveness, Elderly

## Abstract

Disease due to *Streptococcus pneumoniae*, the pneumococcus, remains a major source of illness in older persons. Globally, it remains the most important pathogen in respiratory infection deaths.

Conjugated pneumococcal vaccines are used extensively in national pediatric programs, whereas a polysaccharide vaccine is used in all age groups, but mainly in the elderly and for high-risk groups.

Recent data from the Netherlands led to the licensing in many countries of conjugated pneumococcal vaccines for older persons. There are substantial differences in recommendations from various national immunization technical advisory groups, which owe at least as much to differing assessments of available studies as to differences in local epidemiology.

This review examines those differences and proposes a way forward.

## Background

Infections caused by *Streptococcus pneumoniae*, the pneumococcus, may involve a normally sterile site; for example, blood or joint fluid (known as invasive pneumococcal disease (IPD)], or more commonly a local mucosal infection; for example, community-acquired non-bacteraemic pneumonia (CAP). Pneumococcal infection remains a major source of illness in the elderly. Globally, across all age groups, pneumococcus remains the most important pathogen in deaths due to respiratory infections [1]. Data on IPD cases are relatively robust in many countries, but for CAP the magnitude of the pneumococcus’ contribution is poorly understood.

Two types of vaccine are available and in widespread use in many countries. Conjugated vaccines (PCVs), covering 7 (Prevenar 7® Pfizer, 7vPCV), 10 (Synflorix® GlaxoSmithKline), or 13 serotypes (Prevenar 13 Pfizer, 13vPCV), are used extensively in national pediatric programs, whereas a polysaccharide vaccine containing 23 serotypes (Pneumovax23® Merck, 23vPPV) is used mainly in the elderly and for high-risk groups.

Neither the 7-valent nor the 10-valent conjugated vaccines are commonly used in current immunization programs; therefore, these vaccines are not considered further in this review. Both vaccines contain a subset of serotypes in 13vPCV.

Recent data from the Netherlands Community-Acquired Pneumonia Immunisation Trial in Adults (CAPiTA) led to the licensing in many countries of conjugated vaccine for older persons and also assessment of the role of both vaccines in this age group [2]. However, debate about the effectiveness of vaccines (particularly against CAP), the duration of their effectiveness, the presence of hypo-responsiveness when 23vPPV and a PCV are given in sequence, and herd protection induced by pediatric programs resulting in disease reduction in older persons, has led to various vaccination program recommendations.

There are substantial differences in the recommendations from various national immunization technical advisory groups (NITAGs), which the authors believe owes at least as much to differing assessments of available studies as to differences in local epidemiology.

This review examines those differences and proposes a way forward.

### Burden of pneumococcal disease in the elderly

Assessing data for CAP of any cause and, more specifically, pneumococcal CAP is challenging—there is often no surveillance mechanism in place and published studies have used various combinations of diagnostic tests including blood culture, urinary antigen test, and sputum culture. Additionally, some historical studies used a pneumolysin test that no longer is regarded as diagnostic due to its poor specificity [3].

A review of national databases from 2004 to 2012 and of published studies in Australia set the pneumococcal pneumonia hospitalization rate in those aged ≥65 years at 274/100,000 population, or 20% of all CAP hospitalisations. GP visits for pneumococcal CAP averaged 455 per 100,000 per year. Hospitalizations for IPD in 2012 were only found to be 19/100,000; thus pneumococcal CAP hospitalization rates were 15-fold higher than for IPD and the costs to the healthcare system were determined to be around 30 fold higher [4].

Similarly, a three-year Canadian Serious Outcomes Surveillance (SOS) Network review found 23% of all-cause pneumonia admissions to be due to pneumococcus with a higher percentage in older patients and patients with a higher disease burden and mortality [5]. The number of admissions due to pneumococcal CAP was 13-fold higher than the rate of IPD admission.

### Trends in the burden of disease

#### Is the vaccine- type IPD burden decreasing as a result of herd immunity?

An Australian review of IPD trends in non-indigenous elderly people showed an ongoing substantial decrease in IPD due to serotypes found in the 7 V conjugated vaccine since its introduction in 2004. A similar trend was evident after only about 3 years of use of 13vPCV (against the additional 6 serotypes), and further decline continues. Conversely, likely as a result of serotype replacement, the IPD proportion attributable to 23v–non13v serotypes is increasing, with a rise from 19% to 27% of IPD in Australia [6]. Studies in other countries have confirmed the impact of pediatric conjugated vaccine programs on vaccine-type (VT) IPD rates in older persons [7–9].

A significant proportion of the residual 13vPCV IPD currently seen is due to serotype 3; neither 13vPCV nor 23vPPV appear to have much effectiveness against this serotype. Studies in many countries have shown both poor vaccine effectiveness (VE) and no, or low, impact on disease rates with this serotype [7–9]. In the Andrews review [10] of 23vPPV, the VE against serotype 3 was −23% (95% CI, −85% to −19%).

A meta-analysis of the indirect effects of conjugated vaccines found the mean time taken to attain a 90% reduction in VT IPD due to 7vPCV serotypes was 8.9 years, and 9.5 years for the additional serotypes in 13vPCV, but not in 7vPCV [11].

#### Is the vaccine-type CAP burden decreasing as a result of herd immunity?

While data are more limited and somewhat inconsistent for CAP, a decline in VT 13vPCV CAP is also expected from the childhood program and has already been seen in unvaccinated young adults and older persons in some studies.

A cohort study of non-bacteremic pneumococcal pneumonia cases in adults in Nottingham described a 30% reduction of the proportion of 13vPCV serotypes within 3 years of the switch from 7vPCV to 13vPCV in the childhood program, having already seen an 88% decrease in CAP due to serotypes in 7vPCV [12]. A Dutch review demonstrated a decline in pneumococcal CAP due to 7vPCV serotypes over a 5-year period, from 28% of cases to 7% of cases [13]. In the United States (US), an assessment of the impact of childhood 7vPCV, using the Nationwide Inpatient Sample database, found an annual reduction in pneumonia hospitalisations of 168,000, with the majority of these in older persons [14].

*Both invasive and non-invasive disease rates due to serotypes covered in the childhood 13vPCV program are declining in older persons*.

### Vaccine effectiveness against community-acquired pneumonia

Published estimates place the burden of disease due to CAP (based on hospitalizations) as at least an order of magnitude greater than that due to IPD. Thus the effectiveness of both vaccine groups against CAP is important in the comparative assessment of the two vaccines, even if the burden of disease is decreasing due to serotypes in 13vPCV vaccine.

VE data against CAP with 13vPCV is available from the CAPITA study, which was a randomised controlled trial (RCT). The study found a VE of 45% against VT pneumococcal CAP, 22% against all-type pneumococcal CAP and 5% against all-cause CAP [2].

Reviews of available studies, including RCTs and observational studies, have come to differing conclusions about 23vPPV effectiveness against pneumococcal CAP. The variation occurs in both the estimated VE and the duration of effectiveness. The differences in outcomes of these meta-analyses are due to important variations in study inclusion criteria and variations in the quality and focus of the studies reviewed.

Two of the studies [15, 16] included in some analyses only measured all-cause CAP, thus biasing the observed VE towards no effect. Additionally, in the study by Ortqvist et al. [17] (VE of −18%) the diagnosis of pneumococcal pneumonia was made on the detection of serum antibodies against pneumolysin using poorly validated in-house enzyme-linked immunosorbent assay (ELISA) methods, which was later shown to have poor specificity.

*Clinical studies and review documents have variously ascribed impacts of 23vPPV against pneumococcal CAP from no effect through to around 50% in many studies. While the studies are beset with methodological challenges and difficult to compare, the weight of evidence from the ‘better’ studies suggests that the attributable VE is non-zero and somewhere in this range. Protection against all cause CAP with both conjugate and polysaccharide vaccine types is similar and low, around 5%*.

The observational studies in favour of 23vPPV being effective against VT CAP include a multi-centre Japanese prospective cohort study, which found 33% VE against VT CAP, 27% against all pneumococcal CAP and 2% against all CAP [18]; and a Spanish cohort study that analysed for receipt of vaccine in the last 5 years and found higher VE rates, namely, 48% against pneumococcal CAP and 25% against all cause CAP [19]. A prospective RCT that analysed receipt of 23vPPV in high-risk residents of Japanese nursing homes also found a significant reduction in both pneumococcal pneumonia and all-cause CAP [20].

Supporting a duration of protection with 23vPPV against CAP out to 5 years, Vila-Corcoles found a VE of 46% for pneumococcal CAP analysing subjects who were vaccinated up to 5 years previously [21]. Meta-analytical reviews in favour of 23vPPV effectiveness include a German study [22] allocating a VE against pneumococcal CAP of 64% in clinical trials with follow up of 2.5 years, and 48% in cohort studies followed up to 5 years. A Portuguese review [23] found the range of VE against pneumococcal CAP hospitalisations to be between 32% and 51%, with lower protection if vaccination was given more than 5 years previously. The derived VE against all-cause CAP hospitalisation was 10.2%. A Canadian review focussing on all-cause CAP derived a VE of 4% for trials, 17% for cohort studies and 7% for case control studies and described it as similar to the results from CAPITA, which assessed 13vPCV [24].

The 2013 Cochrane review of 23vPPV vaccine effectiveness against pneumococcal pneumonia derived a pooled VE of 54% (CI 16–75%) but this review included some older PPV formulations with higher antigen contents per serotype [25]. The studies also included many young adults who have stronger immune responses. No benefit was found against CAP for adults in developed countries. The Cochrane review is currently under revision.

Other studies have found no effect of 23vPPV on pneumonia rates. A recent very large study in 152,000 healthy adults demonstrated no vaccine effect on pneumonia incidence [26]. However, this study was conducted over a very short time frame and found no cases at all of pneumococcal CAP. Another retrospective study also concluded that 23vPPv did not affect pneumonia hospitalization rates in the elderly [27].

### Cost effectiveness

The assessment of the cost effectiveness of pneumococcal vaccines in older persons is critically affected by: 1) the estimate used for herd protection produced by the conjugated childhood program against CAP; 2) the estimate used of VE for 23vPPVagainst pneumococcal pneumonia; 3) the VE for all-cause CAP (for each vaccine); and 4) the duration of effectiveness allocated to each vaccine.

A British review, influenced heavily by the herd protection estimates, suggested that for 13vPCV to be cost effective the vaccine price needed to be negative [28]. In Germany, the decision made by their NITAG to not recommend 13vPCV and to continue using 23vPPV was based on the recent systematic review and meta-analysis by Falconhurst et al. [22], and the cost effectiveness review by Kuhlmann [29]. Again, herd protection was a critical factor, with the 23vPPV effectiveness on pneumococcal CAP a major source of uncertainty. The National Advisory Committee on Immunization in Canada also did not recommend the use of 13vPCV in persons ≥65 years of age.

When making the decision in the US to use both conjugate and polysaccharide vaccines sequentially for 3 years and then review the use of 13vPCV in 2018 [30], the Advisory Committee on Immunization Practices (ACIP) relied in part on a preliminary analysis using a probabilistic model involving a single cohort of persons aged 65 years. This demonstrated that “adding a dose of 13vPCV to the current 23vPPSV recommendations for adults aged ≥65 years would prevent approximately 12,000 cases of CAP over the lifetime of a single cohort of persons aged 65 years … [but] the expected benefits of 13vPCV use among this cohort will likely soon decline to an annually estimated 4,500 cases of CAP averted among persons aged ≥65 years” [31].

A review of the evidence assessed in making this decision highlighted the changing epidemiology of pneumococcal disease and the need to evaluate the utility and effectiveness of the new strategy [32].

### The way forward

The burden of pneumococcal infections remains significant and more effective prevention could be achieved. Both conjugated and polysaccharide vaccines appear to be equally ineffective (around 4–5% VE) against all-cause CAP, in part because of a low contribution of pneumococcal CAP to all-cause CAP but also the increase in non-VT pneumococcal disease being seen.

While some data exists to suggest that 23vPCV is not effective against pneumococcal CAP, enough recent studies suggest that VE with 23vPCV could even be as high as 50% and the duration of effectiveness may stretch to 5 years. There is no case to cease 23vPPV programs in older persons and, despite some hypo-responsiveness concerns, a case can be made for revaccination of older persons with chronic conditions every 5 years. Disease rates increase with increasing age and revaccination is likely to produce long-term antibody levels consistent with primary vaccination [33]. The 5-year interval minimises the increase in self-limiting adverse events seen with revaccination [34].

The case for the additional use of 13vPCV is harder to make on a cost basis. Strong data supports the very significant reduction in VT disease due to 13vPCV in older persons after pediatric conjugate vaccine programs, thereby reducing the cost effectiveness of 13vPCV use for the elderly. An opportunity exists now to await the upcoming US review of using both vaccines sequentially to clarify some of the uncertainties.

Current coverage rates of 23vPPV in older person programs need to be increased. It is likely that the increase in disease in the elderly due to the 11 serotypes contained in 23vPPV (but not in 13vPCV) relates not only to replacement infection but also inadequate coverage rates with 23vPPV.

Where governments can afford to use both vaccines for the elderly (as the US has done), better disease prevention may be achieved. Most importantly, vaccines with a broader coverage and duration of protection are needed.

## Conclusion

Most national advisory bodies have recommended continuation of a polysaccharide program for older persons without the addition of a conjugated vaccine to their program. Monitoring of the burden of disease in different countries over the next few years will be important in providing better data for decision making.
